# Phosphatidylinositol-4,5-Bisphosphate Binding to Amphiphysin-II Modulates T-Tubule Remodeling: Implications for Heart Failure

**DOI:** 10.3389/fphys.2021.782767

**Published:** 2021-12-23

**Authors:** Junlan Zhou, Neha Singh, Chloe Monnier, William Marszalec, Li Gao, Jing Jin, Michael Frisk, William E. Louch, Suresh Verma, Prasanna Krishnamurthy, Elsa Nico, Maaz Mulla, Gary L. Aistrup, Raj Kishore, J. Andrew Wasserstrom

**Affiliations:** ^1^Department of Medicine (Cardiology), Feinberg Cardiovascular Research Institute, Northwestern University Feinberg School of Medicine, Chicago, IL, United States; ^2^Institute for Experimental Medical Research (IEMR), Oslo University Hospital, Oslo, Norway; ^3^K. G. Jebsen Cardiac Research Center, University of Oslo, Oslo, Norway

**Keywords:** BIN-1, heart failure, PIP2—phosphatidylinositol-4,5-bisphosphate, T-tubule disruption, Ca^2+^ transients

## Abstract

BIN1 (amphyphysin-II) is a structural protein involved in T-tubule (TT) formation and phosphatidylinositol-4,5-bisphosphate (PIP2) is responsible for localization of BIN1 to sarcolemma. The goal of this study was to determine if PIP2-mediated targeting of BIN1 to sarcolemma is compromised during the development of heart failure (HF) and is responsible for TT remodeling. Immunohistochemistry showed co-localization of BIN1, Cav1.2, PIP2, and phospholipase-Cβ1 (PLCβ1) in TTs in normal rat and human ventricular myocytes. PIP2 levels were reduced in spontaneously hypertensive rats during HF progression compared to age-matched controls. A PIP Strip assay of two native mouse cardiac-specific isoforms of BIN1 including the longest (cardiac BIN1 #4) and shortest (cardiac BIN1 #1) isoforms as well human skeletal BIN1 showed that all bound PIP2. In addition, overexpression of all three BIN1 isoforms caused tubule formation in HL-1 cells. A triple-lysine motif in a short loop segment between two helices was mutated and replaced by negative charges which abolished tubule formation, suggesting a possible location for PIP2 interaction aside from known consensus binding sites. Pharmacological PIP2 depletion in rat ventricular myocytes caused TT loss and was associated with changes in Ca^2+^ release typically found in myocytes during HF, including a higher variability in release along the cell length and a slowing in rise time, time to peak, and decay time in treated myocytes. These results demonstrate that depletion of PIP2 can lead to TT disruption and suggest that PIP2 interaction with cardiac BIN1 is required for TT maintenance and function.

## Introduction

Transverse tubules (TTs) are membranous invaginations primarily associated with mammalian ventricular myocytes. These inward projections of the external sarcolemmal membrane bring voltage-gated calcium channels on the surface membrane into proximity with the sarcoplasmic reticulum (SR), the main intracellular Ca^2+^ store within the myocyte. Ca^2+^ influx across the sarcolemma activates Ca^2+^-sensitive ryanodine receptors in the SR promoting a synchronous cell-wide Ca^2+^ release via the phenomenon of calcium-induced calcium release (CICR), ultimately leading to myocyte contraction. Many studies have demonstrated TT loss in ventricular myocytes during heart failure (HF) in small animal models such as mice (Louch et al., 2006) and rats (Lyon et al., 2009; Wasserstrom et al., 2009) as well as in larger mammals such as dogs (He et al., 2001; Balijepalli et al., 2003), pigs (Louch et al., 2004; Heinzel et al., 2008), and humans (Louch et al., 2004; Lyon et al., 2009; Crossman et al., 2011). Furthermore, work from several investigators has demonstrated that the loss of TTs may be responsible for the development of diastolic and systolic dysfunction (Song et al., 2006; Wei et al., 2010; Shah et al., 2014) making it of far greater importance to understand the mechanisms of normal TT formation as well as of TT remodeling during HF.

The mechanisms underlying normal TT formation and maintenance are poorly understood. A growing body of evidence suggests that the cardiac isoform(s) of amphiphysin-II (BIN1) may play a more central role in TT formation in skeletal and heart muscle. Isoform 8 of BIN1 has the ability to form TTs in skeletal muscle cells (Lee et al., 2002) and mutation of the amphiphysin-II gene was associated with a disrupted TT network (Razzaq et al., 2001). Other studies showed an association of BIN1 with muscular dystrophy as well (Nicot et al., 2007; Fugier et al., 2011). BIN1 knockdown also produces a cardiomyopathy (Muller et al., 2003), making this protein a potential mediator for TT remodeling during the development of HF.

The role of a phosphatidylinositol-4,5-bisphosphate (PIP_2_) interaction with Bin1 in TT formation and maintenance is established skeletal muscle but unclear in the heart. Skeletal BIN1 contains a unique sequence domain of 15 amino-acids called Exon10 (Ramjaun and McPherson, 1998). The primary structure of Exon10 is very similar to a binding motif for PIP2 (Martin, 1998) and Lee et al. (2002) showed that Exon10 has a high specificity to associate with PIP_2_. In addition, PIP_2_ binding to BIN1 opens the SH3 domain to protein-protein interactions, providing a means by which BIN1 could assemble a structural backbone for TT formation, raising the question of whether BIN1 localization and subsequent sarcolemmal TT formation might in fact be PIP_2_-dependent. Although little is known about changes in PIP_2_ during the early stages of HF, there is a clear reduction of PIP_2_ in end-stage HF (Tappia et al., 1999; Ziegelhoffer et al., 2001). This could reduce targeting of BIN1 to the sarcolemmal membrane and thereby disrupt maintenance of the TT structure. However, an important study by Hong et al. (2014) found that none of the four identified cardiac BIN1 (cBIN1) isoforms in mouse heart contained the critical exon 10 (re-named Exon 11 in mouse heart). The implication of these results was that this exon *could* not bind PIP2 and that therefore TT formation was independent of PIP2-BIN1 interactions. To explore the possible interaction between PIP2 and BIN1 and the role of PIP2 in TT formation, through a pivotal role of PIP_2_ in BIN1 organization, we investigated the idea that PIP2 is important for cardiac TT formation and maintenance and that the depletion of sarcolemmal PIP_2_ results in TT degeneration.

## Materials and Methods

### Animal and Human Tissue Samples

#### Animal Studies

All experiments using animals were performed according to a protocol approved by Institutional Animal Care and Use Committee of the Northwestern University and according to the NIH guidelines for animal use. Hearts were obtained from Sprague Dawley rats (aged 4–6 months) and age-matched Wistar Kyoto and spontaneously hypertensive rats (SHR) ranging between 2 and 20 months of age. Rats were injected with 750 units of heparin 5 min prior to anesthesia induced by an injection mixture (80 mg/kg:10 mg/kg) of ketamine and xylazine. When the rats were fully anesthetized, the heart was quickly excised and processed for myocyte isolation, protein extraction or tissue fixation.

#### Human Samples

Normal human tissue samples were obtained from The Gift of Hope of Illinois. All samples were obtained from healthy organ donors whose hearts were considered unsuitable for transplantation. Upon harvest, these hearts were immediately placed in cold cardioplegia solution. Tissue samples were then perfused with 2% paraformaldehyde for subsequent processing. The protocol for tissue use was reviewed by the Institutional Review Board of Northwestern University (exemption 4) and informed consent was obtained from families of organ donors before tissue was collected for research purposes.

All reasonable requests for original data analyses will be fulfilled upon written request to the corresponding author in support of transparency and reproducibility requirements. Full details of all methods are provided in the online Supplementary Material.

## Results

### Molecular Interactions Between Amphiphysin-II Isoforms and Phosphatidylinositol-4,5-Bisphosphate

#### Amphiphysin-II, Phosphatidylinositol-4,5-Bisphosphate, and Phospholipase-Cβ1 Are Distributed Along Transverse Tubules

**FIGURE 1:**
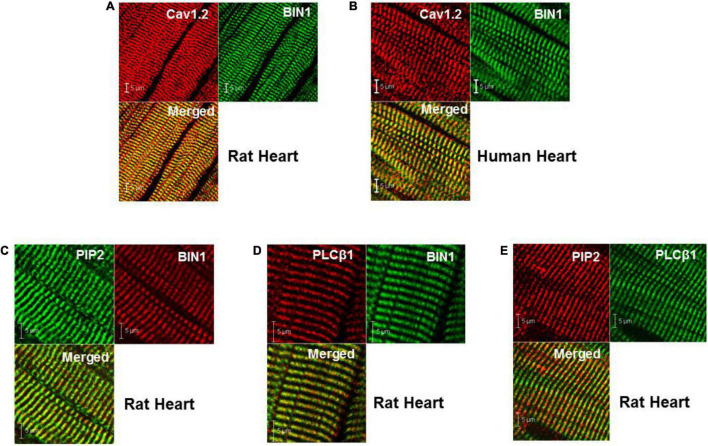
Cellular localization of BIN1, PIP2, and PLCβ1 at TTs in both rat and human heart. **(A,B)** Co-localization of BIN1 with Cav1.2 in rat and human ventricle. **(C–E)** PIP2 and PLCβ1 are also localized at t-tubules in rat heart. Pixel size was 0.02–0.04 μm/pixel and all images were obtained with the pinhole adjusted to Airey units = 1.

Immunohistochemistry co-localization studies of BIN1 with Cav1.2, the L-type Ca^2+^ channel protein present at a high density in TTs, indicated that isoforms of cardiac Bin1 (cBIN1) co-localized with Cav1.2 along the TTs in both normal rat (Wistar Kyoto or WKY rats) and human ventricular tissue (Figures 1A,B). The distribution pattern of PIP2 and phospholipase-Cβ1 (PLCβ1) in rat ventricular tissue showed that both co-localized at TTs with cBIN1 (Figures 1C–E). These observations demonstrate that all four proteins are present exclusively in the TTs, raising the possibility that an important relationship exists between Ca1.2, cBIN1, PIP2, and PLCβ1.

#### Reduction in Phosphatidylinositol-4,5-Bisphosphate Levels During the Progressive Development of Heart Failure in Spontaneously Hypertensive Rats

**FIGURE 2:**
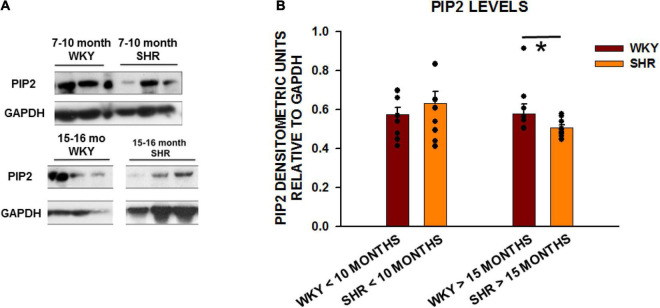
PIP2 levels are reduced with age in SHRs in comparison to control WKY rats. **(A)** Expression of PIP2 from left ventricular tissue in two different age group of animals with young animals < 10 months of age (WKY *n* = 8 rats; SHR *n* = 6 rats) and old animals > 15 months (WKY *n* = 8 rats; SHR *n* = 9 rats). **(B)** Graph of relative protein expression normalized to GAPDH. Values indicated mean ± SEM here and in all subsequent experiments. Comparisons were made using an unpaired *t*-test. *p < 0.05.

SHRs develop severe cardiac structural and functional changes as they age (Shah et al., 2014). We next investigated whether the levels of BIN1 and PIP2 change in left ventricular tissue from SHRs from young (<10 month) and old (>15 month) rats with WKY rats as the normal controls. We found no change in BIN1 protein expression in SHRs as a function of age (Supplementary Figure 1). However, when we also measured PIP2 levels in these same cardiac samples in young (2–10 months old) and aged (15–20 months old) (Figure 2A), we found that PIP2 levels were unchanged in the younger animals but decreased significantly in old SHRs in comparison to WKYs (Figure 2B).

#### Amphiphysin-II Overexpression Induces Sarcolemmal Tubular Extensions in HL1 Cells

**FIGURE 3:**
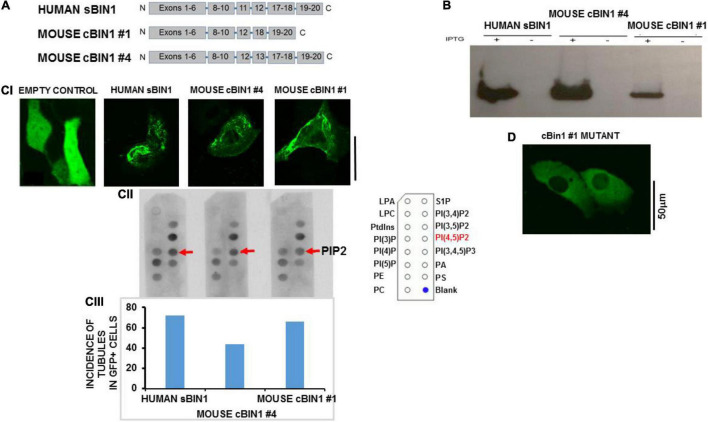
*In vitro* model of HL1 cells shows tubules after BIN1 overexpression. **(A)** Gene organization for two splice variants of mouse cardiac muscle BIN1 as well as human skeletal muscle BIN1. **(B)** The expression of BIN1 proteins induced by isopropyl-b-D-thiogalactoside (IPTG) in bacteria. **(C)** The interaction between purified Bin1 and PIP2. **(CI)** Tubule formation in Hl-1 cells 48 h after overexpression with each of the Bin1 constructs. The inset shows that no tubules formed in the overexpression of empty vector. **(CII)** PIP Strip assays were performed using BIN1 antibody. Representative results from 3 separate experiment are shown and all PIP strip results were confirmed in 1–2 additional strips for each protein. The template describing location of dots for all phospholipids is shown in Supplementary Figure 3. **(CIII)** Incidence of tubule formation induced by over expressed BIN1 in HL1 cells for each construct. No tubules were formed in cells with empty vector. (*N* = 48 cells/3 cultures for each including empty vector). **(D)** Example of an HL-1 cell over-expressing a construct with triple mutations of Lys164Glu/Lys165Glu/Lys166Glu in a loop segment of the N-BAR coiled-coil domain completely abolished tubule formation. No tubulation was found in any GFP-positive cells (*N* = 36 cells/3 cultures). Note that the vector contains a GFP tag so Bin1 can be easily visualized. Pixel size was 0.11 μm/pixel and all images were obtained with the pinhole adjusted to Airey units = 1.

To investigate the interaction between cBIN1 and PIP2, we cloned two splice variants of mouse cBIN1 (#1 and #4) as well as human skeletal muscle BIN1 (isoform 8) in pET6xHN. These cBIN1 splice variants exclude mouse exon11 (previously exon10) and either include 13 and 17 (full-length variant #4) or exclude these two exons (variant #1, as shown in Figure 3A). cBIN1 expression was confirmed in bacteria by western blotting (Figure 3B). PIP Strip assays further confirmed an interaction between purified cBIN1 and PIP2 (Figure 3CII). Interestingly, both cBIN1 and human skeletal muscle BIN1 interacted with other phosphoinositides, including phosphatidylinositol-4,(or 3, or 5)-phosphate and PIP2, [PI(3,5)P_2_] [PI(3,4)P_2_] [PI(3,4,5)P_3_], but not with phosphatidylinositol (PI), phosphatidylserine (PS), phosphatidylcholine (PC), and phosphatidic acid (PA).

Next, the ability of each isoform to induce tubule formation was examined. We developed an *in vitro* model that permitted the study of tubule formation and biochemical signaling pathways that are responsible for both TT loss and maintenance. For purposes of comparison, we performed di-4-ANEPPs staining in intact normal rat ventricle which showed a well-organized and dense TT network (Supplementary Figure 2A). In contrast, staining of HL-1 cells showed a lack of tubulation. We then assayed for BIN1 expression using Western blot analysis and found no protein expression for BIN1 in HL-1 cells (Supplementary Figure 2B), confirming that HL-1 cells had no tubules and lacked BIN1. Overexpression of both isoforms of cBIN1 in HL1 cells induced tubule formation as did human sBIN1 (Figure 3CI). Moreover, all isoforms induced tubule formation in a very large percentage of GFP+ cells, ranging from 72 to 44% (Figure 3CIII). These results indicate that both the shortest and longest splice variants of cBIN1 are capable of inducing tubule formation and that all are capable of binding PIP2 similar to their skeletal counterpart despite the absence of exon 11. Furthermore, binding of PIP2 to cBIN1 does not require the presence of exons 11, 13, or 17 and must reside at another site or sites on the cardiac isoforms. Note that HL-1 cells transfected with empty vector failed to produce any tubules (Figure 3CI).

In order to explore the role of PIP2 binding and tubule formation, we then created a mutant variant of cBIN1 #4 by substituting three lysine residues with negatively charged aspartic acid residues in a loop segment of the N-BAR coiled-coil domain. When this Lys164Glu/Lys165Glu/Lys166Glu mutant was transfected to HL-1 cells, there was no observable tubule formation (Figure 3D), suggesting the structural importance of positive charges in the loop segment in anchoring cBIN1 lattice to phospholipids in promoting tubule formation.

#### Depletion of Phosphatidylinositol-4,5-Bisphosphate Levels by Pharmacologic Intervention

To determine if depletion of PIP_2_ underlies TT remodeling, we performed a series of experiments with pharmacological agents known to decrease cellular PIP_2_ levels by either interfering with PIP2 production or increasing its catalysis. Wortmannin inhibits phosphoinositol-4 kinase (PI4K), an enzyme required for PIP_2_ synthesis (Brown et al., 2007; Albert et al., 2008). Inhibition of PI4K interferes with PIP_2_ regeneration and thus reduces its membrane levels. Endothelin-1 (Et-1) is the endogenous activator of a receptor-mediated signaling cascade involving a Gαq activation of several isoforms of phospholipase C (including sarcolemmal PLCβ1) by which PIP_2_ is hydrolyzed to diacylglycerol and inositol-3-phosphate (DAG and IP_3_). Finally, m-3M3FBS is a direct activator of PLCβ (and subsequent PIP2 hydrolysis).

**FIGURE 4:**
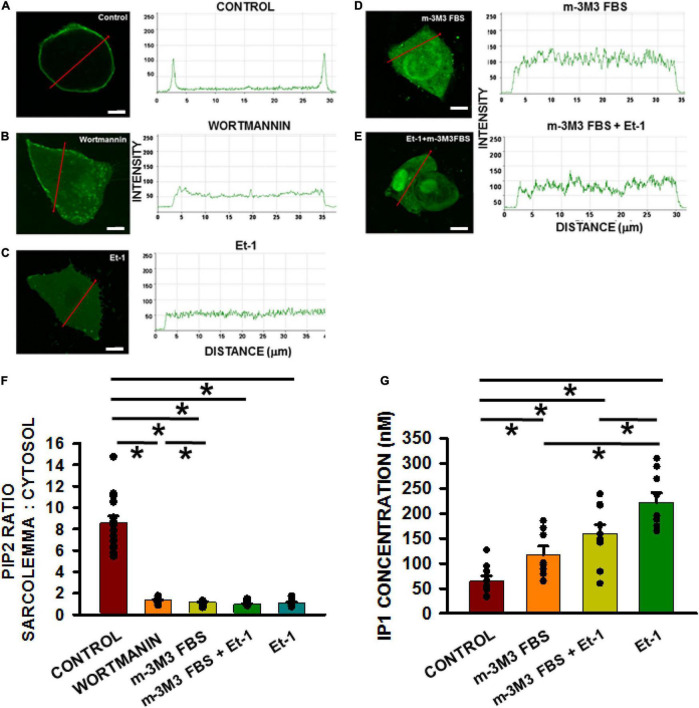
Membrane PIP2 levels decrease significantly in HL-1 cells with active treatments compared to non-treated controls. PIP2 depletion measurements in HL1 cells transfected with the PLCδ1-PH-GFP construct. Quantification measured as the fluorescence intensity change along the red line from membrane to cytosol (**A–E**, scale bar = 5 μm). Pixel size was 0.11 μm/pixel and all images were obtained with the pinhole adjusted to Airey units = 1. Bar graph **(F)** shows results of controls (*n* = 15 wells) compared to cells pre-incubated with wortmannin (*n* = 13), m-3M3FBS (*n* = 12 wells), Et-1 (*n* = 12), and combination of Et-1 and m-3M3FBS (*n* = 15 wells) from 3 separate cultures each. **(G)** Measurements of IP1 in different treatment groups. Control *n* = 9 wells; Et-1 *n* = 8; m-3M3FBS *n* = 9; m-3M3FBS+Et-1 *n* = 10 obtained from 3 cultures each. Statistical comparisons were made using a one-way ANOVA in **(F,G)**. *p < 0.05.

To demonstrate that TT loss mediated by these pharmacological interventions was indeed a result of PIP2 depletion/reduction, PIP2 levels were measured following each of these treatments. First HL1 cells were transfected with a PLCδ1-PH-GFP construct that acts indirectly as a PIP2 reporter. PLCδ1-PH-GFP binds with sarcolemmal PIP2 but is released in the cytosol when PIP2 levels decline. We monitored PH-GFP translocation after treating Bin1- cells with either 15 μM wortmannin (2 h), 200 nmol/L Et-1 (for 48 h), 30 μm m-3M3FBS (30 μmol/L; for 3 h) or the combination of Et-1 and m-3M3FBS (for 45 min) (Figure 4). In the absence of any pharmacological treatments, PIP2 was localized in the sarcolemma (Figure 4A). In contrast, the construct was translocated to the cytoplasm after treatment with wortmannin, Et-1, m-3M3FBS and the combination of Et-1 and m-3M3FBS (Figures 4B–F). Note also that PIP2 depletion was more effective via direct activation of PLCβ1 with m-3M3FBS than with wortmannin. These results support the notion that each of these agents causes PIP2 depletion from the sarcolemma.

To confirm that the treatments indeed cause PIP2 depletion of PIP2 through activation of PLCβ1, the enzyme activity was measured directly using an ELISA assay kit for IP1 which is an easily measured downstream metabolite of PIP2 and IP3 since the latter is extremely difficult to measure directly. We found that IP1 was significantly increased in response to wortmannin (*p* < 0.05), Et-1 (*p* < 0.05), m-3M3FBS (*p* < 0.05), and the combination of Et-1 and m-3M3FBS (*p* < 0.05) (Figure 4G). Our results thus suggest that increased PIP2 metabolism and subsequent PIP2 depletion can be responsible for tubule disruption and strongly indicate that PIP2 is required for TT maintenance.

### Effects of Phosphatidylinositol-4,5-Bisphosphate Depletion on Transverse Tubule Density and Excitation-Contraction Coupling in Adult Rat Ventricular Myocytes

#### Phosphatidylinositol-4,5-Bisphosphate Depletion Causes Transverse Tubule Disruption in Isolated Adult Rat Ventricular Myocytes

In order to extend our observation that PIP2 plays a key role in tubule disruption in a cell culture system to adult ventricular myocytes, we investigated the effects of pharmacological depletion of PIP2 on TTs in isolated adult rat ventricular myocytes (ARVMs). We have previously described our measurements of TT organization, measured as organization index (OI), in adult myocytes using di-4-ANNEPS staining (Aistrup et al., 2013).

**FIGURE 5:**
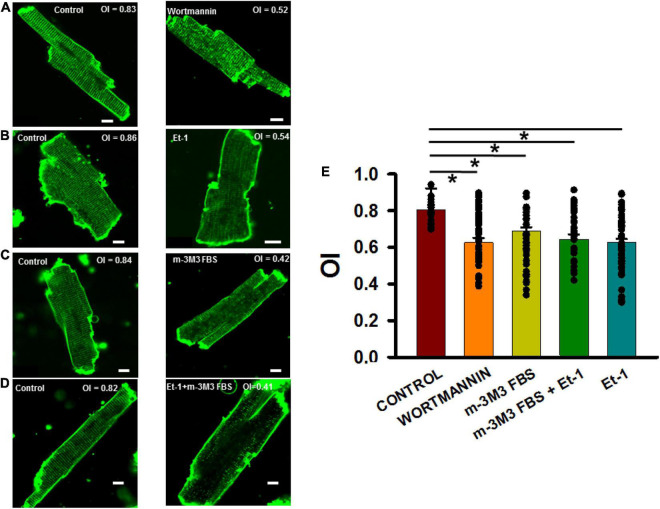
PIP2 depleting agents adversely affected organizational index (OI) of TTs in isolated adult rat ventricular myocytes. **(A–D)** 2-D images of control (left panels) and treated (right panels) myocytes. **(E)** Summary of OI changes untreated cells (35 myocytes in 3 rats) and after treatment with wortmannin (15 μmol/L, 2 h, *n* = 35/3), m-3M3FBS (30μmol/L, 3 h, *n* = 37/5), and Et-1(400 nmol/L) + m-3M3FBS (30 μmol/L, 45 min, *n* = 35/4) and Et-1 (400 nmol/L, 6 h, *n* = 54/4). *p < 0.05 using Dunn’s test following a one-way ANOVA performed on all cells. Pixel size was 0.45 μm with the pinhole adjusted to Airey units = 1.

Figure 5A (left panel) shows a typical ventricular myocyte with TTs that traverse the entire cell width at even spacing of about 2 μm. Severe TT remodeling was observed in cardiomyocytes exposed to wortmannin treatment (Figure 5A, right panel). Similar results were obtained in myocytes treated with Et-1 (Figure 5B), m-3M3FBS (Figure 5C), and in combination (Figure 5D). Figure 5E summarizes these results and demonstrates that all four treatments that deplete PIP2 also significantly reduced OI in rat ventricular myocytes.

**FIGURE 6:**
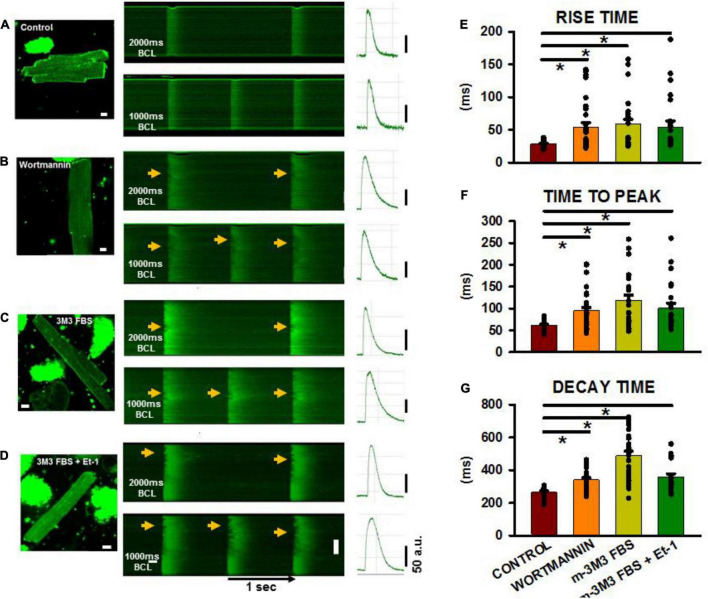
Calcium transients in isolated adult ventricular myocytes after PIP2 depletion. **(A–D)** 2-D images with associated linescan images from treated and non-treated myocytes. Pacing was performed at basic cycle lengths of 2,000 and 1,000 ms. Arrows shows areas of delayed Ca^2+^ release along the cell length in the myocytes. **(E–G)** Shows summaries of rise time, time to peak and decay time from control and treated cells at a pacing cycle length of 1,000 ms. Control (*n* = 18–28 myocytes from 6 rats), wortmannin (27–36/5), m-3M3FBS (27/3), Et-1+m-3M3FBS (*n* = 22–24/3). *p < 0.05 using a Dunn’s test following a one-way ANOVA performed on cells. Pixel size was 0.2 μm with the pinhole adjusted to Airey units = 1.

Finally, we wanted to demonstrate that TT disruption was directly responsible for altered Ca^2+^ release properties in adult myocytes following pharmacologically-induced PIP2 depletion. We and others have demonstrated that progressive heart failure is characterized by TT loss and profound disruption of sarcoplasmic reticulum Ca^2+^ release and reuptake (Louch et al., 2006, 2012; Frisk et al., 2016). Therefore, we measured Ca^2+^ rise times, times-to-peak and decay times in order to determine if they were affected by pharmacological disruption of TTs in a manner similar to that reported to occur following TT disruption in heart failure. Figures 6A–D show longitudinal confocal line scan recordings of Ca^2+^ transients in isolated ARVMs both under control conditions and following treatment with each of the agents inducing PIP2 depletion and TT loss. The 2-D images show representative myocytes in control (Figure 6A) and following treatment with wortmannin (Figure 6B), m-3M3FBS (Figure 6C), and combination of the Et-1 and m-3M3FBS (Figure 6D). Note the high OI in the untreated control and corresponding low values of OI in the treated cells. When we measured Ca^2+^ transients at slow (BCL = 2,000 ms) and rapid (BCL = 1,000 ms) pacing rates in the control myocyte, Ca^2+^ release was rapid and uniform along the entire cell length and the decay of the transient was also rapid at both rates (linescan images in Figure 6A), In contrast, each linescan image from the drug-treated cells showed regions of late release (arrow heads) at the slow rate that were exaggerated during rapid pacing (linescan images in Figures 6B–D). The result is a slowing in overall rise time and decay time of the transients that was exaggerated at the faster rates in each of the cells with poor TT organization, as indicated by the average fluorescence intensity profiles at the right of each image. The summary graphs for the Ca^2+^ transients during rapid pacing (Figures 6E–G) show that the overall rates of Ca^2+^ release and sequestration were delayed in all the drug-treated myocytes compared to untreated cells as indicated by the significant increases in rise times, times-to-peak and decay times of the Ca^2+^ transients.

**FIGURE 7:**
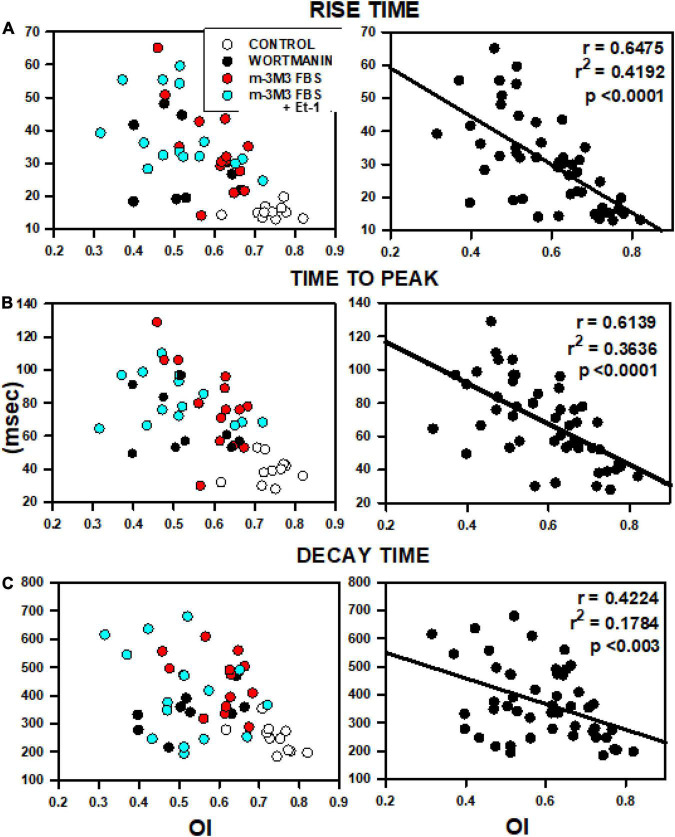
Relationships between OI and rise time **(A)**, time to peak **(B)** and decay time **(C)**. Data were obtained from 5 rats from the analyses presented in Figure 6 including data from control myocytes (open circles), myocytes treated with wortmannin (filled circles), 3M3FBS (red circles) and combination Et-1 + 3M3FBS (cyan circles).

When rise time, time to peak and decay time were plotted for all cells treated with PIP2-depleting agents as a function of mean OI (Figure 7), close inverse relationships were observed, indicative of slowed Ca^2+^ release and removal with decreased OI (Figures 7A–C). The left panels show myocytes that were treated with wortmannin, m-3M3FBS, or the combination of m-3M3FBS + Et-1 and each treatment is individually color-coded. Control cells (open circles) have high OI and are characterized by rapid Ca^2+^ release and recovery. In contrast, treated cells (filled circles) show dramatic slowing in release and recovery properties as OI decreases. There is no obvious departure of any cell group from the overall relationship, suggesting a commonality in mechanism, in this case the decrease in OI. These data suggest that the relationship between OI and each characteristic of Ca^2+^ release or removal occurs along a continuum that is largely independent from the specific treatment involved so that the slowing of release and of removal is reliant on the reduction in OI and not on how it was achieved. This is best demonstrated in the graphs in the right panels in which the linear fit for each overall relationship is shown. Each graph shows a significant reliance of Ca^2+^ release and reuptake on OI that is not related to the specific treatment by which reduced OI was induced. We have previously shown nearly identical results in individual myocytes from aged SHRs (Singh et al., 2017) which strongly suggests the critical role of TT remodeling on Ca^2+^ cycling even in myocytes where TT loss was acutely and artificially induced through pharmacological means but which yields results that are nearly identical to those found in HF. We have previously shown nearly identical results in individual myocytes from aged SHRs (Singh et al., 2017). Taken together, these results demonstrate that TT disruption induced by PIP2 depletion causes morphological and physiological alterations that share a very close resemblance with those found in HF.

## Discussion

### Cardiac Amphiphysin-II Isoforms and Transverse Tubule Formation

Although cardiac TT remodeling and associated dysfunction of Ca^2+^ release have been extensively reported in HF (Balijepalli et al., 2003; Louch et al., 2004, 2012; Song et al., 2006), the processes underlying the formation and maintenance of TTs in general are not known, although we do know that BIN1 seems to be a critical component of TT assembly. In skeletal muscle, this protein has several unique features that make it well-suited for this role, including a natural curvature, a charge profile that allows anchoring in the negatively charged phospholipids in the membrane bilayer, and a PIP2 binding site in Exon 10 that is unique to the muscle (and TT bearing) isoform of the amphiphysin-II protein family. With the exception of Exon 10, nearly all of these properties appear to be shared by the cardiac isoforms of this protein leaving us with an incomplete understanding of these processes in heart.

In cardiomyocytes, Hong et al., 2014 have shown that one of four cBIN1 isoforms in mouse adult cardiomyocyte (BIN1+13+17) was involved in cardiomyopathy and arrhythmia by regulating ion flux and TT morphology (Hong et al., 2014). It has been shown that exon 10 of BIN1 contains a basic amino-acid sequence (RKKSKLFSRLRRKKN) that is important to TT biogenesis in skeletal muscle and the interaction of Exon10 with PIP2 appears to be crucial for localization of BIN1 to TTs in skeletal muscle (Lee et al., 2002). However, our data show that mouse cBIN1 without Exon10/11 still binds a number of phosphoinositides including PIP2. Moreover, overexpressed cBIN1 was capable of tubule formation. These data indicate that Exon 10/11 is not essential for the interaction between cBIN1 and PIP2 for cardiac TT formation. Furthermore, we found that at least one critical PIP2 binding site on cBIN1 is not located at either exon13 or exon17 but does appear to adjoin the BAR domain where its mutation prevented tubule formation. Thus, the current study suggests a novel mechanism that allows cBIN1 and PIP2 to play a crucial role in TT biogenesis. It is also important to note that recent work has also identified the presence of exon 11-containing isoform 8 in rat (Li et al., 2020) but not apparently in mouse (Hong et al., 2014) myocardium. Furthermore, Guo et al. (2021) have shown that at least two different exon 11-containing isoforms 8 and 13 (i.e., Bin1+11 and Bin1+11+17, respectively) are also present in human heart. These results indicate that there are indeed exon 11-containing isoforms in hearts other than mouse that are likely to interact with PIP2 through this motif as well as possible additional binding sites described here that do not rely on the presence of exon 11.

### Phosphatidylinositol-4,5-Bisphosphate, Phospholipase-Cβ1, Amphiphysin-II, and Transverse Tubules

One important finding is the co-localization of PLCβ1, BIN1, and PIP2, at TTs in adult ventricular myocytes. This is likely to be the PLCβ1b isoform since the other minor cardiac isoform (PLCβ1a) is located in the cytoplasm unlike membrane-bound PLCβ1b (Grubb et al., 2008, 2011). TT disruption in HF abolished both the normal striated patterns of Cav1.2 and of BIN1 organization without an overall loss of BIN1 (Supplementary Figure 4). Thus, BIN1 organization is lost in HF without reduced protein levels when PIP2 is no longer available to insure BIN1 membrane insertion and its absence also reduces BIN1 interactions with neighboring proteins. Finally, this co-localization of PLCβ1, BIN1, and PIP2 at TTs also suggests the existence of a signalasome responsible for TT formation and maintenance, supporting the idea that TT remodeling may result from a loss of one or more members of a putative signaling complex.

### Phospholipase-Cβ1 Activity, Phosphatidylinositol-4,5-Bisphosphate Depletion and Transverse Tubules

Hypertension and other cardiovascular diseases that often develop into HF are known to increase levels of endothelin-1 (Miyauchi et al., 1989; Yasuda et al., 1990; Tsuji et al., 1991; Bohm and Pernow, 2007) and angiotensin-II (Gray et al., 1998; Strawn et al., 2000; Molkentin and Dorn II, 2001; Ayabe et al., 2006; Palaniyandi et al., 2009). These factors serve as agonists for a G protein-coupled signaling cascade through Gαq activation which in turn activates several isoforms of phospholipase C, including PLCβ1, found in the sarcolemma. In addition, mRNA and protein levels of PLCβ1 are increased in ischemia (Asemu et al., 2003) and in stroke-prone SHRs as young as 8–10 months there is increased sarcolemmal protein kinase C (PKC) activity (Kawaguchi et al., 1993), suggesting increased PLCβ1 activity. Here, we propose that it is the resulting decrease in membrane PIP2 content itself that has important consequences for TT maintenance and efficient excitation-contraction (EC) coupling.

In order to investigate the role of PIP2, we first focused on the properties of BIN1. Recently, it has been reported that BIN1 serves as an anchor for Ca_*v*_1.2 localization to cardiac TTs, thus promoting specific microtubule-induced trafficking of new Ca_*v*_1.2 molecules to the T-tubular membranes (Hong et al., 2010). This is an important observation because it demonstrates that BIN1 may insure the localization of proteins crucial for EC coupling to the TT and thus serve as an important candidate around which to build the structural and functional TT unit. With this in mind, we measured BIN1 protein expression in SHRs in comparison to normal WKY rats from ages 2–20 months and found no change in BIN1 levels with HF progression but rather extensive disorganization of this protein. We have previously reported that SHRs show significant abnormalities in systolic and diastolic function by 12–17 months as a sign of advancing HF (Shah et al., 2014). In the same study we showed that SHRs show TT disorganization in some myocytes as early as 9-months and that by the age of 17-months a significant reduction in TT organization is apparent in nearly all myocytes. Our current results show that PIP2 levels were significantly decreased during HF development in SHRs especially after the age of 12 months which is consistent with the onset of TT loss during HF development.

### Amphiphysin-II Overexpression and Tubulation in HL-1 Cells

In this study, we wanted to explore the properties of BIN1 in a cell system that did not have inherent expression of this protein or TTs. This approach allowed us to examine the properties of BIN1 in tubule formation and how it would respond to PIP2 depletion. We used mouse HL1 cells which do not express BIN1 and found that the exogenous overexpression of all isoforms of BIN1 induced tubulation. We then used this model system to demonstrate that PIP2 depletion could be accomplished in any number of ways including activation of PLCβ or by inhibition of PIP2 synthesis via PI4-kinase (PI4K), both of which subsequently were found to abolish TTs in adult ventricular myocytes. The translocation of the membrane PIP2-binding reporter PLCδ1-PH-GFP from sarcolemma to cytosol following these pharmacological treatments suggested that tubule loss was a result of sarcolemma PIP2 depletion. These results suggest that not only does BIN1 have the potential to promote tubulation but it also requires the presence of membrane PIP2 to do so.

### A Molecular Model for the Role of Cardiac BIN1 in Transverse Tubule Formation

**FIGURE 8:**
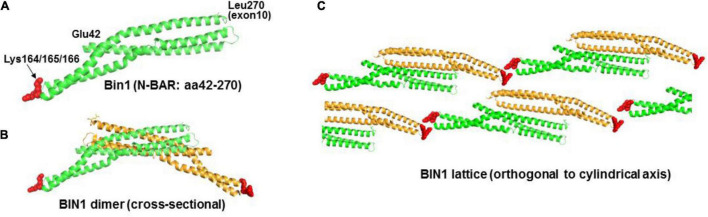
Proposed model of BIN1 in T-tubule formation. **(A)** Crystal structure of N-BAR of BIN1 (PDB ID: 2FIC) consists of a coiled-coil domain. The short loop segment between helix 2 and 3 has three lysine residues from amino acid 164 to 166 (red). **(B)** Two BIN1 N-BARs (one in green color and the other in orange color) form a “banana”-shaped curve through their lateral interactions. **(C)** In T-tubule formation, BIN1 N-BAR domain oligomerizes to form a patterned lattice (modeled based on F-BAR domain) around the lipid bilayer.

We postulated that the N-BAR domain alone, without the Exon 10/11 sequence such as in cBIN1 isoforms, can still bind PIP2. In fact, the concept of structurally related F-BAR domains capable of binding phosphoinositol was studied previously by Frost et al. (2008) Mutagenesis analysis of the F-BAR domain of FBP17 protein showed that combined Lys56Glu and Arg104Asp substitutions resulted in an absence of tubulation. Considering that Lys56 and Arg104 are residuals of the α-helices that may be directly involved in the integrity of the helical structures or in maintaining coil-to-coil interactions, the argument of these cationic residues being the anchoring sites for phospholipid interactions remains inconclusive. The N-BAR domain of BIN1 also forms oligomeric lattices with narrower tubules than those induced by F-BAR proteins (Frost et al., 2009). We noticed a triple-lysine motif (Lys164-Lys165-Lys166) in a short loop segment between two helices and subsequently demonstrated that mutations of these cationic residues abolished tubule formation. This cationic motif is located at the distal tips of an N-BAR dimer and the mutant is not expected to affect the integrity of the BAR domain structure (Figures 8A,B). When we modeled the BIN1 lattice in self-assembly during tubule induction and highlighted the pattern of tip-to-tip locations in Figure 8C, we found an alternative scenario of unified T-tubule formation through a charge-based BIN1-to-phosphoinositol interaction. These theoretical results support the idea of a novel mechanism by which PIP2 may induce BIN1 oligomerization with resulting TT formation and maintenance.

### A Role for Cardiac BIN1 and Phosphatidylinositol-4,5-Bisphosphate Interaction in Cardiac Transverse Tubule Formation

In order to extend our observations in the HL-1 cell culture system to native cardiac myocyte structure and function, we repeated the PIP2 depletion experiments in native ventricular myocytes and found a similar dramatic reduction of TT organization following pharmacological PIP2 depletion. PIP2 depletion induced by either PI4K inhibition or PLCβ1 activation had virtually identical effects to disrupt TT organization to that reported for HF myocytes. As postulated earlier, PLCβ1 activation, as a member of putative BIN1, PIP2, and PLCβ1 complex located in the TTs, may lower endogenous PIP2 membrane levels in a manner similar to what we observed in culture. Thus, PIP2 depletion via PLCβ1 hydrolysis may eventually disrupt BIN1 targeting to plasma membranes where PIP2 ordinarily helps to localize this protein causing the cBIN1/PIP2 complex to lose its ability to maintain TTs and inducing TT remodeling in HF.

### Effect of Transverse Tubule Loss in Failing Heart

HF myocytes show a reduction of both the rate and magnitude of SR Ca^2+^ release that contribute to poor contractile performance at the subcellular and cellular levels and ultimately to systolic dysfunction in the heart. Slow rise times and times to peak in myocytes after PIP2 depletion is consistent with the finding that PIP2 loss disrupts TTs thereby reducing Ca^2+^ influx through L-type Ca^2+^ channels and impairing CICR from SR. This point is important since cellular regions lacking TTs then have large distances across which remote TT Ca^2+^ influx must diffuse to reach the SR membrane (and so-called orphaned RyRs) in order to induce CICR compared to areas with normal TTs. Thus, instead of a simultaneous activation of contraction throughout the cell, individual cell regions without local TTs will do so with a delay resulting in a dyssynchronous contraction, reducing contractile efficiency. Another feature of altered Ca^2+^ cycling is slow reuptake (Beuckelmann et al., 1992; Balke and Shorofsky, 1998; O’Rourke et al., 1999; Gomez et al., 2001; Wasserstrom et al., 2009) and we observed a slower decay time of Ca^2+^ transients in these cells, affecting the synchrony of relaxation. The primary reason for this slowing is that the time to peak release is slowed and, furthermore, there is increased heterogeneity of release along the cell length. This variability in release presumably occurs where orphaned RyRs must wait for CICR from nearby release units to increase local Ca^2+^ concentration and activate release at those sites which no longer have nearby L-type Ca^2+^ current to trigger release. Because of the delay in release, there is a slowing in rise time and time-to-peak as seen both in our experiments with pharmacologically-induced PIP2 depletion and TT loss (Figure 6) as well as in HF (Louch et al., 2006, 2012; Song et al., 2006; Shah et al., 2014; Singh et al., 2017). The result is also a slowing in recovery of the Ca^2+^ transient as different regions of the cell go through delayed release and resulting removal of cytoplasmic Ca^2+^.

Sarcolemmal PIP2 may also help localize other sarcolemmal proteins including K^+^ channels and the Na-Ca exchanger, thereby affecting cytosolic Ca^2+^ homeostasis through other mechanisms (Hilgemann and Ball, 1996; Huang et al., 1998). The progressive amplification at the multicellular level of this intracellular phenomenon probably contributes to the impairment of overall myocardial function observed in HF.

### Limitations

It is possible that the immunohistochemistry results may be affected by some experimental artifact that could bias our interpretation of apparent co-localization between these proteins. However, we took great care to insure that Cav1.2 was used as an indicator of the location of TTs and that any subsequent localization of the other proteins was aligned with this accepted standard for the localization of TTs. The close alignment between Cav1.2 and Bin1 (Figures 1A,B) as indicated in the merged overlap image cannot be explained as resulting due to chance without invoking explanations that are clearly unrealistic. Thus, although there may be some minor experimental artifacts that might reduce the accuracy of co-localization, such as poor alignment in the z-plane due to the point spread function inherent in confocal microscopy, it is highly unlikely that our results and interpretation are inaccurate overall.

Overall, our work provides extensive evidence that (1) PIP2 binds to Bin1, which has been a highly controversial issue in the heart, (2) Bin1 isoforms are capable of inducing tubule formation, and (3) PIP2 depletion causes loss of TTs in the heart in a manner that mimics the TT remodeling that occurs in HF. On the other hand, we have not provided definitive proof that the PIP2 depletion is directly responsible for TT loss. Our current methodologies do not permit us to measure this interaction directly but our results provide critical new evidence that PIP2 depletion occurs in HF and that pharmacological PIP2 depletion is associated with the loss of TTs in cardiac myocytes. The hope is that, in the future, we will be able to observe this relationship with higher precision in order to provide definitive proof of this reliance of TT remodeling on PIP2 regulation during the development of cardiac disease.

### Clinical Implications

Our data suggest that BIN1 localization at the sarcolemma is required for TT formation and that PIP2 functions as a membrane-targeting and stabilization element for the formation of TTs. These observations are crucial to our understanding of TT remodeling in HF since reduced sarcolemmal PIP2 levels would then be expected to reduce BIN1 targeting to the sarcolemma and hence tubulation. This finding suggests a novel mechanism for TT formation and maintenance, both normal and abnormal. Approximately 80% or more of all L-type Ca^2+^ channels are present on TTs, which in turn are critical for the synchronous release of Ca^2+^ in myocytes and normal EC coupling in heart. Our observation that both delayed Ca^2+^ release and reuptake are closely related to TT organization due to PIP2 depletion strongly suggests that the loss of PIP2 may be the mechanism for TT disruption during disease development and the resulting defects in Ca^2+^ cycling and progressive decline in myocardial mechanics. Our study provides important new insights into the mechanistic understanding of TT organization and its implications in Ca^2+^ dynamics in normal and diseased states.

Based on our results, it might be possible to interfere with degenerative TT remodeling by targeting pathways leading to PIP2 loss. A better mechanistic understanding of the roles of BIN1 and PIP2 in TT remodeling may be important to the development of novel therapeutic interventions aimed at the restoration of this putative phospholipid-protein interactive complex both for the prevention of and the reversal of HF once it has developed.

## Data Availability Statement

The original contributions presented in the study are included in the article/Supplementary Material, further inquiries can be directed to the corresponding author/s.

## Ethics Statement

The studies involving human participants were reviewed and approved by the Northwestern University Institutional Review Board, Northwestern University, Evanston, IL. Written informed consent for participation was not required for this study in accordance with the national legislation and the institutional requirements. The animal study was reviewed and approved by the Institutional Animal Care and Use Committee, Northwestern University, Evanston, IL.

## Author Contributions

JZ, NS, JW, and JJ designed and performed experiments, analyzed the data, wrote parts of the manuscript, and approved the final version. CM, WM, and LG designed and performed the experiments, analyzed the data, and approved the final version. MF, WL, PK, SV, RK, and GA designed the experiments and approved the final version. EN and MM analyzed the data and approved the final version. All authors contributed to the article and approved the submitted version.

## Conflict of Interest

The authors declare that the research was conducted in the absence of any commercial or financial relationships that could be construed as a potential conflict of interest.

## Publisher’s Note

All claims expressed in this article are solely those of the authors and do not necessarily represent those of their affiliated organizations, or those of the publisher, the editors and the reviewers. Any product that may be evaluated in this article, or claim that may be made by its manufacturer, is not guaranteed or endorsed by the publisher.
