# Supplementary material for: Phosphatidylinositol-4,5-Bisphosphate Binding to Amphiphysin-II Modulates T-Tubule Remodeling: Implications for Heart Failure
Source: Front Physiol. 2021 Dec 23;12:782767. doi: 10.3389/fphys.2021.782767 (PMC8733645; doi:10.3389/fphys.2021.782767)
Supplement: Supplementary file 1 [file Data_Sheet_1.docx]

**SUPPLEMENTAL MATERIAL**

Expanded Methods

**Cell culture and Transfection**: Myocytes from the mouse atrium HL1 cell line were cultured in Claycomb medium supplemented with 10% fetal bovine serum, 0.1 mmol/L norepinephrine, 2 mmol/L L- glutamine and penicillin/streptomycin (100U/mL). A GFP-tagged transfection-ready DNA ORF clone of human BIN1 (transcript variant 8) (Origene, RG220616) and PLCδ1-PH-GFP (kindly provided by Dr. Ming Zhao, Northwestern University) were overexpressed in HL1 cells. Approximately 3-5x10^4^ cells were plated on glass cover slips on 24 well plates 24 hrs prior to transfection. When cells were approximately 70-80% confluent, a transient transfection was achieved by the use of lipofectamine LTX and Plus Reagent (Invitrogen, A12621/15338-030). GFP tagged empty control vectors were used for control experiments. Confocal imaging was performed 24 to 48 hours later using the intracellular GFP expression to validate the successful transfection of individual cells.

**Immunohistochemistry (IHC) and western blots:** For immunohistochemistry, rat hearts were perfused with sterile cold PBS followed by 10% formalin for 10-15 min. The ventricles were then fixed in 10% phosphate buffered formalin for 24 hours. After fixation hearts were sectioned into base, mid and apex and paraffin blocks were prepared. Prior to IHC, tissue was de-parafinized then washed with PBS, fixed with 4% paraformaldehyde for 10 minutes and then permeablilized with 0.05% Triton-X. Tissue blocking was done using blocking solution (3% bovine serum albumin, 10% Horse serum and 0.2% Triton X-100) for 1 hour at room temperature. Samples were then incubated at 37^o^C for 2 hrs with primary antibodies and 45 min with the appropriate secondary antibody conjugated to Alexa-488 or Alexa 594 (Invitrogen). Samples were covered with mounting media (Invitrogen) and overlaid with coverslips. Afterwards, paraffin blocks were prepared and 4 µm thick sections were examined under a confocal microscope. Immunostaining with Cav1.2 antibody was used to locate T-tubules (Millipore mouse monoclonal antibody MAB13170).

Protein lysates from tissue or cells were prepared in RIPA cell lysis buffer (pH 7.4) containing 1% CHAPS and supplemented with complete mini protease inhibitor (Roche). Supernatants were collected after centrifugation (14000g) for 20 minutes at 4^o^C and used for western blot analysis. Cell lysates were boiled in Laemmle sample buffer (0.5 mol/L Tris-HCl [pH 6.8], 10% SDS, 10% glycerol, 4% β-merceptoethenol and 0.05% bromophenol blue) at 80^o^C for 15 minutes. Equal amounts of protein (40µg) were loaded in each well on SDS-PAGE (10%, Biorad) for separation and then electro-transferred on PVDF membranes. After BSA or 5% milk was applied to block non-specific binding, the membrane was probed with mouse monoclonal antibodies to either PIP2 (PIP2 2C11, Santa Cruz) or BIN1 (Amphiphysin- II 2F11, Santa Cruz) overnight at 4^o^C. The following day the protein blots were developed with anti- mouse secondary antibody. GAPDH was used as loading control. Protein band visualization was achieved using ECL-Plus reagent (Pierce; Thermo Fisher Scientific, Rockford, IL, USA) and the band intensity was quantified by densitometric scanning with ImageJ software (NIH, Bethesda, MD).

**Purification of skeletal and cardiac isoforms of BIN1:** Full-length mouse cardiac BIN1 (#1 and #4) and human skeletal muscle BIN1 were expressed from pET6xHN in E. coli BL21 (DE3). Bacteria were grown in LB medium overnight at 37°C and then innoculated to a 50ml culture by dilating the overnight culture1/20 with LB medium until an OD of 0.7. Bacteria were grown in LB medium for 5 hours at 37°C after IPTG (1mM) was added to induce protein expression. After harvesting, cells pellets were resuspended in 1X Equilibration buffer with lysozyme (0.75mg/ml) ml for 20min at 4°C and spun at 12,000 g for 20min at 4°C to remove cell debris. Supernatant was analyze by SDS-PAGE to detect BIN1 protein by western blotting with BIN1 antibody. Supernatant was also loaded onto a His60 Ni Gravity column, incubated for 1 hour at 4°C, and washed with His60 Ni equilibration buffer. Target protein was eluted with elution buffer. Purified BIN1 was concentrated, aliquoted and stored at −80°C.

**PIP Strip assay:** PIP strip membranes facilitate the analysis of phosphoinositide protein interaction by protein-lipid overly assay. PIP strip membranes from Echelonwere Biosciences were used. BIN1 protein was purified by His60 Ni resin. The assay was performed as follows: the strip membrane was blocked with 3% fatty acid free bovine serum albumin (BSA) in TBS-T( 0.1% v/v Tween-20) for 1 hour at room temperature. Membrane was incubated with 0.5ug/ml purified BIN1in TBST 3% BSA for 1hour at room temperature by gently agitation. The washing of membrane was performed three time with TBST for 10min each time. Membrane was incubated with anti-BIN1 antibody in TBST 3% BSA for 1hour at room temperature. For the detection of bound protein, Pierce ECL western blotting substrate was used.

**Adult rat ventricular myocyte isolation:** Calcium tolerant adult rat ventricular myocytes (ARVM) were obtained from Sprague-Dawley rats (240 to 260 g) by enzymatic isolation. Hearts were retrograde-perfused on a Langendorff apparatus with buffered Tyrode’s Solution containing (in mmol/L): 140 NaCl, 5.4 KCl, 1 MgCl_2_, 0.4 NaH_2_PO_4_, 10 glucose, 10 N-2- hydroxyethylpiperazine N′-2-ethanesulphonic acid (HEPES) for 5 minutes at 37°C before the perfusion solution was switched to one containing 0.3 to 0.6 mg/mL of Liberase TH (Roche) in 66 µmol/L Ca^2+^. After 20 minutes of perfusion, the left ventricle was dissected out and minced in a petri dish. The tissue was then transferred to a glass tube containing Ca^2+^-free Tyrodes solution containing 2% BSA where it was gently triturated for 1 minute. The resulting cell suspension was then filtered through a 200 µm nylon mesh into a new tube, washed with 10 ml of Ca^2+^-free Tyrodes and allowed to settle. The over-lying cell-free solution was then removed and replaced three times with Tyrodes having increments of Ca^2+^ at 0.1, 0.2 and 0.5 mmol/L. This made the cells “calcium-ready” to promote their survival during their subsequent exposure to the physiological Ca^2+^ levels of the recording solution (1.8 mmol/L).

**Pharmacological modification of PLCβ1 activity**: Post-transfected HL1 cells were treated with several agents reported to effect the activation of the primary ventricular isoform (β1) of phospholipase C (PLCβ1) or its downstream pathways. These agents and their treatment durations included endothelin-1 (Et-1; Sigma) at a concentration of 200 nmol/L for 6 hours, m-3M3FBS (Sigma; 30µmol/L for 3 hours), o-3M3 FBS (Santa Cruz; 30 µmol/L for 3 hours), and wortmannin (Millipore; 5nmol/L or 15µmol/L for 2 hours). Combination treatments included Et-1(400nM) + m-3M3FBS (30µmol/L) for 45 minutes and Et-1(400nmol/L) + o-3M3FBS (30µmol/L) for 45 minutes. In addition the isolated ARVMs were treated with the same reagents over the same time periods at a concentration of 400nmol/L for 6hrs, with the exception of the Et-1 treatment. In order to visualize t-tubules in BIN1-overexpressed HL1 cells and rat myocytes following the above treatments, cells were incubated in 10µM RH-237 (Assay Biotechnology) prior to confocal imaging. **Inositol monophophate (IP1) measurement for PLCβ1 activity:** An IP1-Elisa (Cisbio Bioassays, Bedford, MA) kit was used to measure PLCβ1 activity in HL1 cells. Cells were plated at a density of 70,000 cells in 24-well plates and grown until they reached 80-90% confluence. The cells were incubated in a buffer media provided with the kit to which was added the pharmacological activators of PLC at the concentrations and times mentioned above. All procedures were followed according to the manufacturer’s instructions.

**Confocal imaging of T-tubules and calcium transient analysis:** Single photon confocal microscopy was performed with a 488 nm Argon laser using a Zeiss LSM510 confocal microscope to observe HL1 cells expressing the GFP-BIN1 construct. The triple Lys164Glu/Lys165Glu/Lys166Glu mutant was generated through site-directed mutagenesis using the following primer pair: 5’-cttcaaactgccgaagaggaggatgaagccaaaattgccaagcct and 5’-tttggcttcatcctcctcttcggcagtttgaagggactcgtagtg. Tubules were observed using the membrane dye di-4-ANEPPS. All imaging was performed with a 40X water immersion objective. Adult rat ventricular myocytes were treated with 8 µmol/L di-4-ANEPPS for 15 minutes and then washed before microscopic observation. The quantitative measurement of t-tubule organization was performed using fast Fourier transform (FFT) of T-tubule organization and data are presented as an organizational index (Aistrup et al. 2013). Z-stacks of myocyte T-tubules were analyzed by MATLAB with the outer sarcolemma excluded from analysis.

Ca^2+^ transient measurements were performed in rat myocytes treated with 15µmol/L Fluo-2 AM and 2.5 µmol/L pluronic acid (20%) for 20 mins and then washed with fresh Tyrode’s solution containing 1.8mmol/L Ca^2+^. Cells were stimulated at either a basic cycle length (BCL) of 2000 ms or a rapid CL of 1000 ms and Ca^2+^ transients were recorded along the longitudinal axis of each cell. Calcium transient measurements included time-to-peak, rise times and decay times. Time to peak was defined as time from the initiation of Ca^2+^ release to maximal Ca^2+^ value at the peak of the transient. Rise time was measured as the time difference between Ca^2+^ release from 10% to 90% of peak Ca^2+^. Decay time was measured as the time difference between 90% of peak Ca^2+^ to 10% of that value during the recovery from Ca^2+^ release. These values were then correlated with the quantitative cellular t-tubule organization in the same cells.

**Molecular Modeling:** In our proposed model, the T-tubule is “sculpted” by BIN1 BAR domains. A pair of these BAR domains form a banana-shaped structure that shapes the curvature of the T-tubule membrane. Hundreds of these dimeric BAR domains form a lattice of spiraling rows that surrounds the membrane tube. The interactions between BAR domains and membrane lipids are maintained through electrostatic charge-based attractions, with positively charged BAR domain residues interacting with negatively charged phospholipids. Based on cryo-EM study (Frost et al) and molecular dynamics (Ying et al) of related N-BAR domains, we modeled the formation of BIN1 BAR domain in its lattice configuration. The loop segment consisting of amino acids 164-166 of BAR domain between helices 2 and 3 carries strong positive charges in its interaction with PIP2. These interactions anchor BIN1 at the membrane surface for efficient membrane bending for tube formation.

References:

Aistrup, G. L. *et al.* Inhibition of the late sodium current slows t-tubule disruption during the progression of hypertensive heart disease in the rat. *Am J Physiol Heart Circ Physiol* **305**, H1068-1079, doi:10.1152/ajpheart.00401.2013 (2013).

Frost A, Perera R, Roux A, Spasov K, Destaing O, Egelman EH, De Camilli P, Unger VM. Structural basis of membrane invagination by F-BAR domains. Cell 132:807–817, 2008.

Ying Yin, Anton Arkhipov, and Klaus Schulten. Simulations of membrane tubulation by lattices of amphiphysin N-BAR domains. *Structure*, 17:882-892, 2009.

**Statistics:** Statistical analysis was performed using Sigma Plot 11.0 and the Graph Pad Prism software. Statistical differences between data groups were calculated by the Fisher Exact test, an unpaired t test or a 1-way ANOVA. Statistical significance between experimental groups was accepted when p<0.05. All data are presented as mean ± SEM.

Supplemental Results


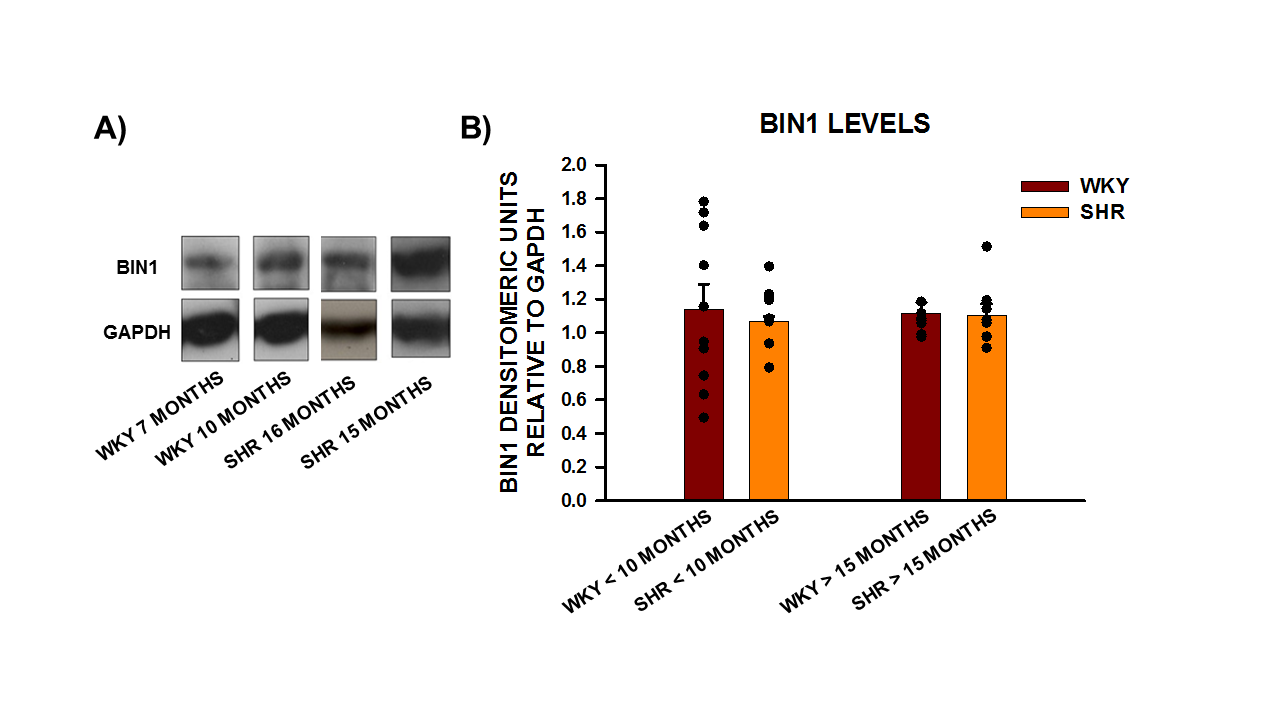


**Supplemental Figure1:** BIN1 expression in young (<10month) and aged (>15month) WKY and SHR rats.


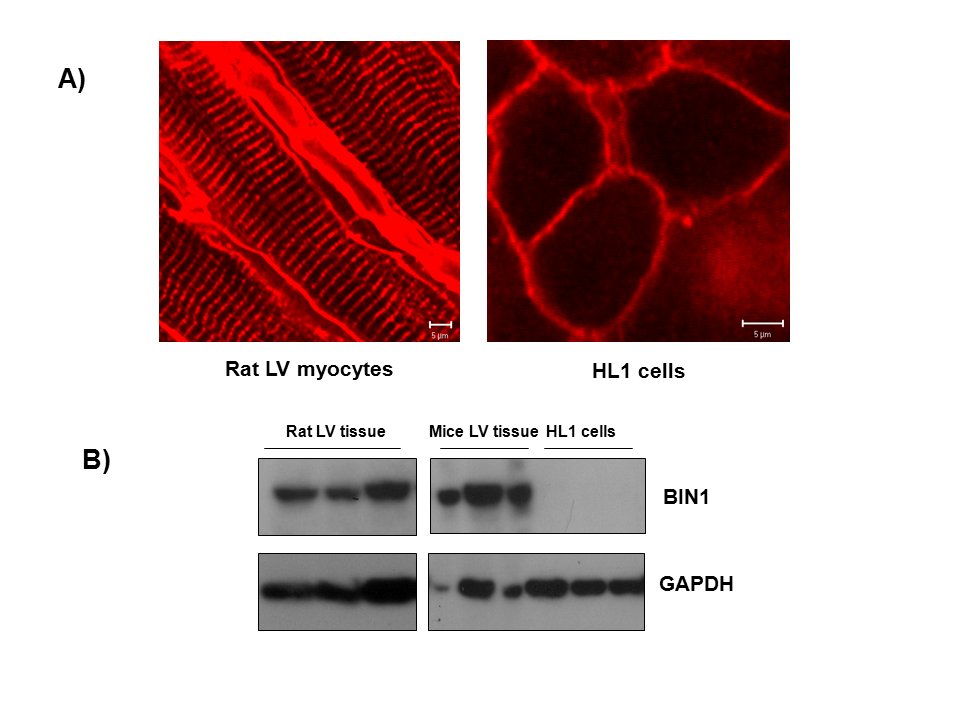


**Supplemental Figure 2:** A) T tubule imaging in rat left ventricular myocytes and HL1 cells. B) Comparative expression on Bin1 in rat left ventricular tissue, mice ventricular tissue and HL1 cells.


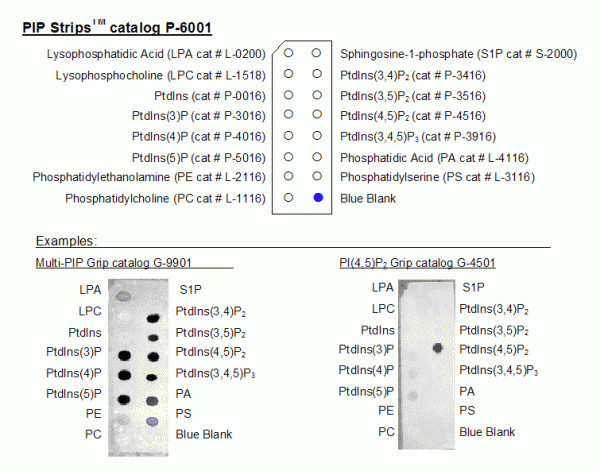


**Supplemental Figure 3:** Template for PIP strip showing all potential phospholipids bound by PLC*β*1 isoforms shown in Figure 3.


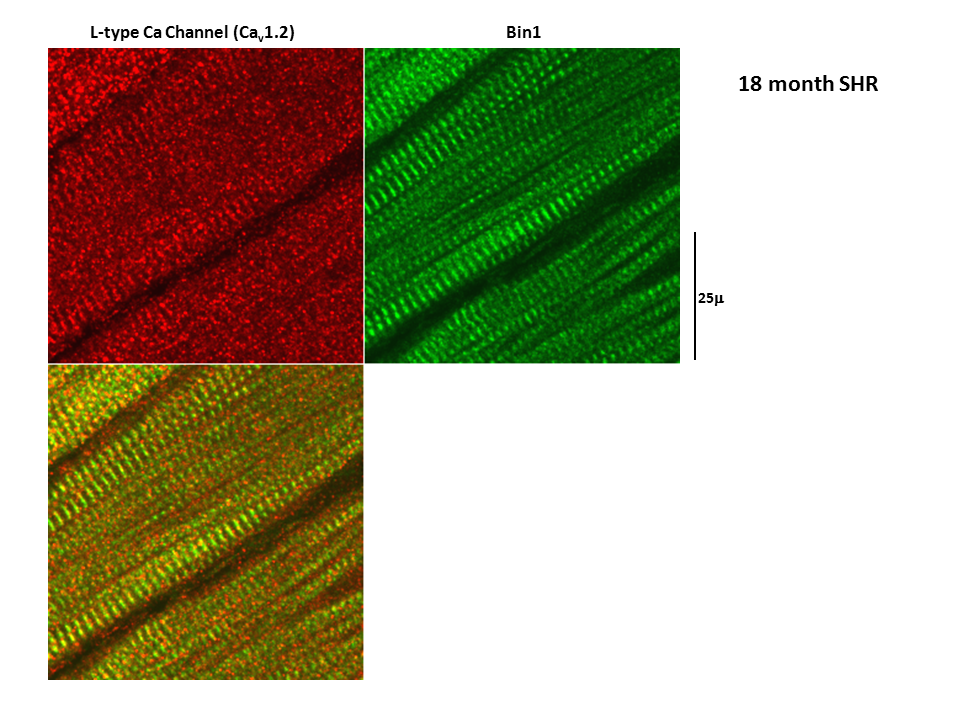


**Supplemental Figure 4:** Loss of T-tubule organization and Cav1.2 in ventricular myocytes in a failing SHR (upper left panel). In contrast, BIN1 organization (upper right panel) is disrupted but not nearly to the same extent as would be expected from T-tubule and Cav1.2 disorganization. More importantly, there is still a great deal of Bin1 protein remaining despite major remodeling of T-tubules.
